# A mutation in Ampd2 is associated with nephrotic syndrome and hypercholesterolemia in mice

**DOI:** 10.1186/1476-511X-13-167

**Published:** 2014-10-31

**Authors:** Joan Helmering, Todd Juan, Chi Ming Li, Mark Chhoa, Will Baron, Tibor Gyuris, William G Richards, James R Turk, Jeff Lawrence, Patrick A Cosgrove, Jim Busby, Ki Won Kim, Stephen A Kaufman, Connie Cummings, George Carlson, Murielle M Véniant, David J Lloyd

**Affiliations:** Department of Metabolic Disorders, Amgen Inc, One Amgen Center Dr, Thousand Oaks, CA 91320 USA; Department of Protein Sciences, Amgen Inc, One Amgen Center Dr, Thousand Oaks, CA 91320 USA; Department of Comparative Biology & Safety Sciences, Amgen Inc, One Amgen Center Dr, Thousand Oaks, CA 91320 USA; UltraPath Imaging, P.O. Box 110294, Durham, NC 27709 USA; McLaughlin Research Institute, 1520 23rd Street South, Great Falls, MT 59405 USA

**Keywords:** LDL, HDL, AMP deaminase, B6, C3, Proteinuria, ENU

## Abstract

**Background:**

Previously, we identified three loci affecting HDL-cholesterol levels in a screen for ENU-induced mutations in mice and discovered two mutated genes. We sought to identify the third mutated gene and further characterize the mouse phenotype.

**Methods:**

We engaged, DNA sequencing, gene expression profiling, western blotting, lipoprotein characterization, metabolomics assessment, histology and electron microscopy in mouse tissues.

**Results:**

We identify the third gene as *Ampd2*, a liver isoform of AMP Deaminase (*Ampd*), a central component of energy and purine metabolism pathways. The causative mutation was a guanine-to-thymine transversion resulting in an A341S conversion in Ampd2. *Ampd2* homozygous mutant mice exhibit a labile hypercholesterolemia phenotype, peaking around 9 weeks of age (251 mg/dL *vs.* wildtype control at 138 mg/dL), and was evidenced by marked increases in HDL, VLDL and LDL. In an attempt to determine the molecular connection between Ampd2 dysfunction and hypercholesterolemia, we analyzed hepatic gene expression and found the downregulation of *Ldlr*, *Hmgcs* and *Insig1* and upregulation of *Cyp7A1* genes. Metabolomic analysis confirmed an increase in hepatic AMP levels and a decrease in allantoin levels consistent with *Ampd2* deficiency, and increases in campesterol and β-sitosterol. Additionally, nephrotic syndrome was observed in the mutant mice, through proteinuria, kidney histology and effacement and blebbing of podocyte foot processes by electron microscopy.

**Conclusion:**

In summary we describe the discovery of a novel genetic mouse model of combined transient nephrotic syndrome and hypercholesterolemia, resembling the human disorder.

**Electronic supplementary material:**

The online version of this article (doi:10.1186/1476-511X-13-167) contains supplementary material, which is available to authorized users.

## Introduction

Hypercholesterolemia is a major risk factor for developing coronary artery disease, the most common cause for heart disease and the leading cause of death worldwide. Coronary artery disease occurs when excess cholesterol in the bloodstream accumulates and forms plaques in the coronary arteries. This narrowing and hardening of the arteries can eventually lead to myocardial infarction. Inherited forms of hypercholesterolemia are present with very high circulating cholesterol levels. The most common type of inherited hypercholesterolemia is familial hypercholesterolemia, which occurs in roughly 1 in 500 people [1]. Known genes related to hypercholesterolemia include low density lipoprotein receptor (*LDLR*) [2], proprotein convertase subtilisin/kexin type 9 (*PCSK9*) [3], apolipoprotein B (*APOB*) [4] and low density lipoprotein receptor adaptor protein 1 (*LDLRAP1*) [5], among others.

A useful tool for discovering other causative genes involved in hypercholesterolemia is mouse genetics. Quantitative trait loci (QTL) analysis has been used to study the genetics of HDL-cholesterol (HDL-C) levels in humans and mice [6]. Phenotype-driven screens such as those using the highly potent mutagen *N*-ethyl-*N*-nitrosourea (ENU) have revealed novel genes and biology [7]. Specifically, genes associated with low HDL-C levels have been found by utilizing ENU [8], as well as genes associated with reduced cholesterol and triglyceride levels [9]. One advantage of ENU screens is that they induce point mutations, thereby allowing the identification of a single candidate gene responsible for the phenotype of interest. ENU screens can also generate an allelic series of mutations that display a range of effects from complete or partial loss of function to exaggerated function, and reveal gene functions in an unbiased manner [10]. We previously performed an ENU screen to identify lines of mice with altered lipid profiles [11]. One line of mice presented undetectable levels of HDL-C, caused by a missense mutation in the ATP binding cassette transporter A1 (*Abca1*) gene. The second line of mice which had very high levels of plasma total cholesterol and HDL-C was mapped to a missense mutation in CCAAT/enhancer binding protein α (*C/ebpα*). A third line of mice was characterized with a reduced penetrance labile hypercholesterolemia phenotype but the underlying mutation had not been identified.

In this study, we identify the mutation as a recessive loss of function missense mutation in the gene encoding adenosine monophosphate deaminase 2 (*Ampd2*). AMPD is the rate-limiting enzyme in the catabolism of adenosine monophosphate (AMP) to uric acid, for the elimination of nitrogenous waste [12]. It is also a component of the purine nucleotide cycle, responsible for the deamination of AMP to inosine monophosphate (IMP), which serves to replenish intermediates in the tricarboxylic (TCA) cycle when energy demands are high. In humans, there are at least four isoforms of AMPD. AMPD1 is the predominant form in skeletal muscle, AMPD2 is widely expressed in non-skeletal tissues, especially the liver, and AMPD3 is found in erythrocytes and is comprised of two isoforms, E1 and E2 [13].

We now complete the physical mapping and define the remaining causative mutation in our hypercholesterolemia mice [11]. We further characterize the *Ampd2* mutant mice as having a transient hypercholesterolemia phenotype that also present with transient nephrotic syndrome (NS). Upon further investigation we find that the dyslipidemia noted in these mice parallels the proteinuria observed and thus represents a novel genetic mouse model of transient NS accompanied with the lipid abnormalities as characterized in the human disease.

## Results

### Identification of a mutation in *Ampd2*

**Figure 1:**
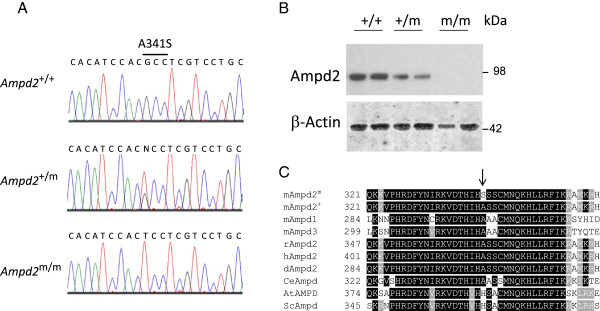
**Identification of a loss-of-function mutation in** ***Ampd2*** **in hypercholesterolemic mice. A)** Sequencing chromatographs of *Ampd2* in *Ampd2* ^+/+^, *Ampd2* ^+/m^ and *Ampd2* ^m/m^ mice. **B)** Liver immunoblot analysis of Ampd2 protein in *Ampd2* ^+/+^, *Ampd2* ^+/m^ and *Ampd2* ^m/m^ mice. **C)** Amino acid conservation of mouse Ampd2 residues 321–361 to Ampd2^+^, mouse Ampd1, mouse Ampd3, rat Ampd2, human Ampd2, dog Ampd2, *Caenorhabditis elegans* Ampd, *Arabidopsis thaliana* AMPD and *Saccharomyces cerevisiae* Ampd*.* Arrow indicates residue 341.

We previously reported [11] the mapping of the mutation for the hypercholesterolemic mice to a 7 Mb region on chromosome 3, with 131 possible candidate genes identified. Forty five were selected for further mutation analysis based on functional relevance. After examining the exons and intronic junctions of these 45 candidate genes by direct sequencing, we identified a guanine-to-thymine transversion resulting in an alanine to serine conversion at amino acid 341 in *Ampd2* (Figure 1A). Using exon capture and high-throughput sequencing we analyzed the region from *D3Mit102* to *rs13477320* in 2 affected animals, three ENU mutations were identified: the A341S missense mutation in *Ampd2*, and intronic mutations in *Magi3* and *Slc6a17* (data not shown). Western blotting illustrated the functional consequence of this mutation on Ampd2 protein expression. Complete loss of Ampd2 protein was observed in the livers of homozygous mutant mice (*Ampd2*^m/m^), with partial loss evident in the heterozygous mice (*Ampd2*^+/m^) relative to wild-type (+/+) (Figure 1B). Alanine 341 was conserved in all animal species including *Caenorhabditis elegans*, but not in plants and fungi (Figure 1C), yet was restricted to a highly conserved region of the protein.

### Lipid characterization of *Ampd2*mice

**Figure 2:**
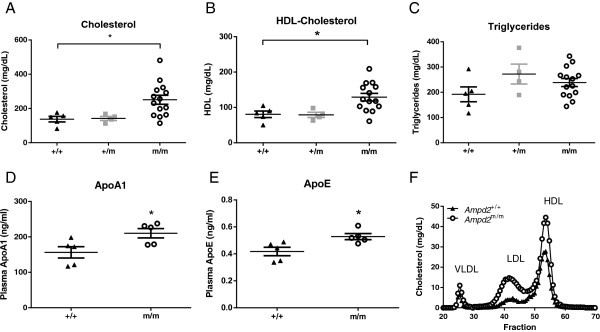
**Lipid parameters of** ***Ampd2*** ^**m/m**^ **,** ***Ampd2*** ^**+/m**^ **and** ***Ampd2*** ^**+/+**^ **mice.** Plasma total cholesterol **(A)**, HDL-C **(B)**, triglyceride **(C)**, ApoA1 **(D)**, ApoE **(E)** levels and FPLC lipoprotein analysis **(F)** of 9-week old mice. Individual mouse values are shown with means ± SEM. Statistical analyses were carried out by one-way ANOVA using a Tukey post test for panels **A**, **B** and **C** and by unpaired two-tailed *t*-tests for panels **D** and **E**; * p <0.05 *vs. Ampd2* ^+/+^.

Lipid parameters were analyzed on *Ampd2*^+/+^, *Ampd2*^+/m^ and *Ampd2*^m/m^ mice at 9 weeks of age (Figure 2). This time point was chosen after the observation was made that these phenotypes were transient in subsequent mouse plasma collections at 9, 11 and 13 weeks [11] and confirmed the previously reported elevated total cholesterol levels in *Ampd2*^m/m^ animals (Figure 2A). Additionally *Ampd2*^m/m^ mice exhibited reduced penetrance, as evidenced by the appearance of some *Ampd2*^m/m^ mice with normal total cholesterol levels (Figure 2A). Levels of HDL-C were also significantly elevated in *Ampd2*^m/m^ mice (Figure 2B), mirroring the total cholesterol values for individual mice. No differences in triglyceride levels between *Ampd2*^+/+^, *Ampd2*^+/m^ and *Ampd2*^m/m^ mice were observed (Figure 2C). Analysis of ApoA1, the major protein component of HDL in plasma, showed a significant elevation in *Ampd2*^m/m^ compared to *Ampd2*^+/+^ mice (Figure 2D) supporting the HDL-C data. Levels of ApoE were also significantly elevated in *Ampd2*^m/m^ compared to *Ampd2*^+/+^ mice (Figure 2E). The elevation of ApoA1 and ApoE in *Ampd2*^m/m^ mice was further evidenced by FPLC analysis of plasma lipoproteins (Figure 2F). As expected *Ampd2*^m/m^ mice were found to have elevated HDL-C levels, in addition very low-density lipoprotein (VLDL) and low density lipoprotein (LDL) levels were increased relative to *Ampd2*^+/+^ mice.

### Hepatic gene expression analysis of *Ampd2*mice

**Figure 3:**
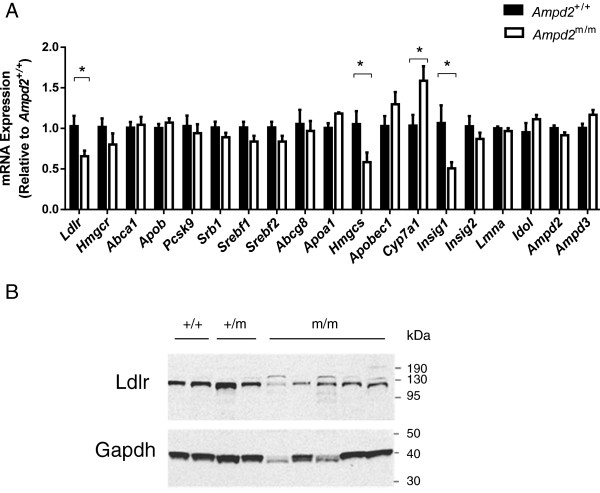
**Gene expression analysis of** ***Ampd2*** ^**m/m**^ **and** ***Ampd2*** ^**+/+**^ **mice. A)** Liver mRNA levels were assessed by quantitative RT-PCR for an array of lipid metabolism genes on fed 9-week old *Ampd2* ^+/+^ and *Ampd2* ^m/m^ mice (n = 5). Statistical analyses were carried out by unpaired two-tailed *t*-tests; * p <0.05 *vs. Ampd2* ^+/+^. **B)** Liver immunoblot analysis of Ldlr protein in *Ampd2* ^+/+^, *Ampd2* ^+/m^ and *Ampd2* ^m/m^ mice.

In an attempt to uncover a pathogenic mechanism for the observed hypercholesterolemia we focused our attention on liver lipid metabolism. An array of lipid metabolism genes were analyzed by quantitative RT-PCR in the livers of *Ampd2*^+/+^ and *Ampd2*^m/m^ mice at 9 weeks of age (Figure 3A). *Ldlr* mRNA was decreased by 36%, which translated to reduced protein levels of Ldlr (Figure 3B), consistent with elevated LDL cholesterol levels (Figure 2F). Transcript levels of 3-hydroxy-3-methylglutaryl-CoA synthase (*Hmgcs*) were also 47% lower. The cholesterol and bile acid synthesis enzyme cholesterol 7 alpha-hydroxylase (*Cyp7a1*) mRNA was increased by 55% in *Ampd2*^m/m^ mice. Insulin-induced gene 1 (*Insig1*) was decreased by 56% in *Ampd2*^m/m^ mice. We confirmed normal expression of *Ampd2*, indicating the mutation had no effect on mRNA stability, whereas it likely resulted in protein instability (Figure 1B). We also confirmed normal expression of the broadly expressed paralog *Ampd3*, suggesting no overlapping compensation. In general it appeared that the changes in gene expression were a consequence of high cholesterol levels, and not causative, prompting us to employ additional technologies.

### Metabolomic analysis of *Ampd2*deficient mice

**Figure 4:**
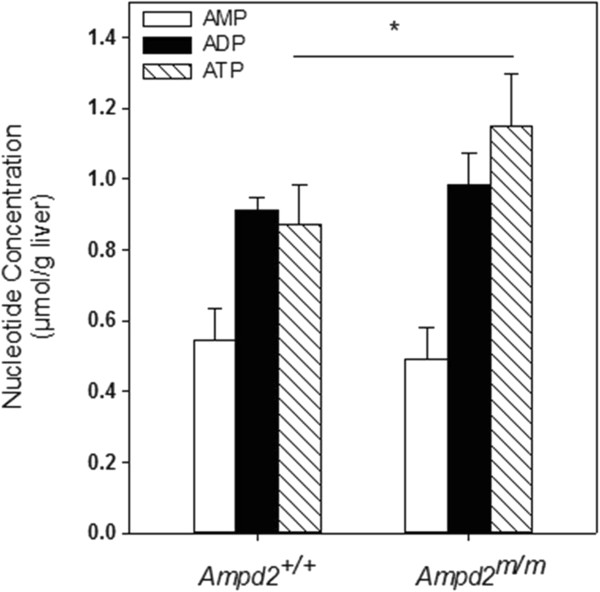
**Hepatic AMP, ADP, and ATP levels in** ***Ampd2*** ^**+/+**^ **and** ***Ampd2*** ^**m/m**^ **mice.** Hepatic AMP, ADP, and ATP levels were determined by HPLC. Open bars represent AMP; solid bars - ADP and hatched bars - ATP. Statistical analysis was carried out by unpaired two-tailed *t*-tests, * p <0.05 *vs. Ampd2* ^+/+^.

We engaged metabolomics to assist in determining the mechanism of hypercholesterolemia in *Ampd2*^m/m^ mice. Liver and plasma from 9-week old *Ampd2*^+/+^ and *Ampd2*^m/m^ mice were collected for metabolite analysis. These analyses confirmed the cholesterol phenotype of the *Ampd2*^m/m^ mice in both plasma and liver samples (Additional file 1: Table S1). Additionally cholesterol esters were elevated up to 5 fold. Allantoin, the terminal metabolite in the purine degradation pathway was significantly reduced in the livers from *Ampd2*^m/m^ mice (Additional file 1: Table S1). The upstream metabolites AMP and ATP were significantly increased in *Ampd2*^m/m^ mice (Additional file 1: Table S1 and Figure 4). Metabolomic analysis also demonstrated significant increases in plasma plant sterols including campesterol and β-sitosterol.

### Comparison of *Ampd2*deficiency on different genetic backgrounds

**Figure 5:**
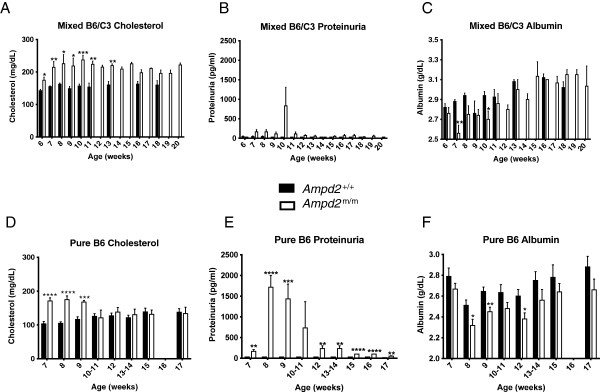
**Temporal hypercholesterolemia and proteinuria in** ***Ampd2*** ^**m/m**^ **mice in mixed B6/C3 and pure B6 genetic background. A and D)** Plasma cholesterol level time course in *Ampd2* ^+/+^ and *Ampd2* ^m/m^ mice. **B and** **E)** Proteinuria level time course in *Ampd2* ^+/+^ and *Ampd2* ^m/m^ mice. **C and** **F)** Plasma albumin levels time course in *Ampd2* ^+/+^ and *Ampd2* ^m/m^ mice on a mixed B6/C3 background and pure B6 background (n = 4-6). Statistical analyses were carried out by unpaired two-tailed *t*-tests; * p <0.05; ** p <0.01; *** p <0.001; **** p <0.0001 *vs. Ampd2* ^+/+^.

Initial gene mapping was carried out by outcrossing the ENU harboring C57BL/6 J (B6) genome to C3.SW-*H2*^b^/SnJ (C3) mice, and all studies presented to date have been using mice from heterozygous matings with this mixed genetic background. A homogeneous genetic background was achieved by backcrossing the *Ampd2*^m/m^ mice to inbred B6 mice to obtain a pure B6 background. *Ampd2*^+/+^ and *Ampd2*^m/m^ mice on a mixed B6/C3 background were compared to those on a pure B6 background with a focus on plasma cholesterol and urinalysis [14]. *Ampd2*^m/m^ mice on both backgrounds exhibited dramatically elevated levels of urinary protein between 7 and 11 weeks of age, most evident in B6 mice (Figure 5B and 5E) than the mixed B6/C3 line, and showing significance throughout the course of the analysis. Hypercholesterolemia was evident in *Ampd2*^m/m^ mice on both genetic backgrounds, with the mixed B6/C3 background showing higher absolute cholesterol levels, whereas B6 mice exhibited a milder but less variable phenotype. Additionally, the mixed background *Ampd2*^m/m^ mice had consistently higher cholesterol levels *vs.* the *Ampd2*^+/+^ mice throughout the course of the study, peaking at 8–10 weeks of age, while the pure B6 *Ampd2*^m/m^ mice had elevated cholesterol levels relative to the *Ampd2*^+/+^ mice only from 7–9 weeks of age (Figure 5A and 5D). Consistent with the presence of proteinuria and NS (see below), both backgrounds of *Ampd2*^m/m^ mice displayed hypoalbuminemia (Figure 5C and 5F), and similarly to cholesterol levels, albumin levels attained statistical significance at a young age for both lines. Together these data in both genetic backgrounds revealed that the proteinuria observed temporally coincided with the cholesterol phenotypes, suggesting interplay between the traits.

### Confirmation of nephrotic syndrome in *Ampd2*^m/m^mice

**Figure 6:**
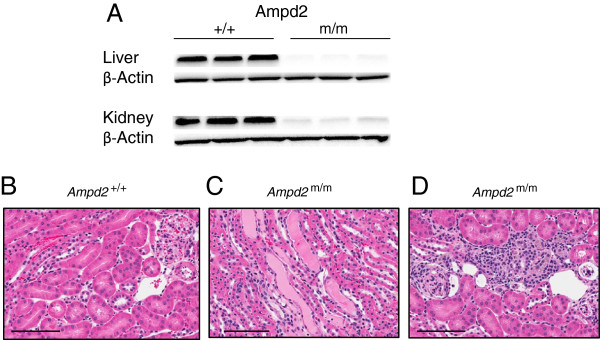
**Ampd2 deficiency and histological features in** ***Ampd2*** ^**m/m**^ **kidneys. A)** Liver and kidney immunoblot analysis of Ampd2 protein in B6 *Ampd2* ^+/+^ and *Ampd2* ^m/m^ mice. **C)** Proteinaceous tubular casts were observed in the kidneys of *Ampd2* ^m/m^ mice. **D)** Interstitial nephritis was observed in the kidneys of *Ampd2* ^m/m^ mice, but not in *Ampd2* ^+/+^ mice **(B)**. Bar = 100 μm.

**Figure 7:**
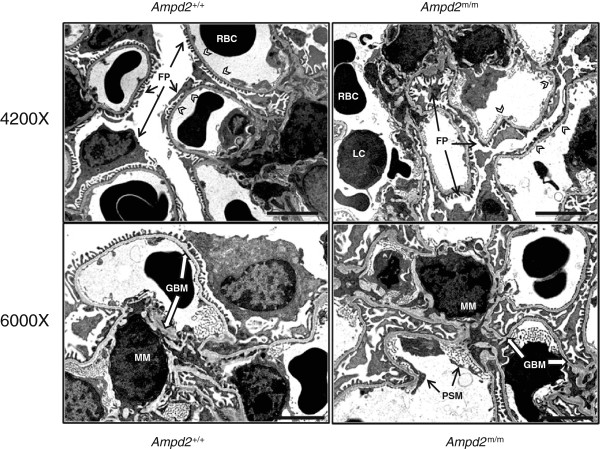
**Electron micropscopic evaluation of the glomeruli from** ***Ampd2*** ^**m/m**^ **mice.** Glomeruli of *Ampd2* ^m/m^ mice showed diffuse effacement, sloughing, and fusing of podocyte foot processes (FP). Extensive blebbing, fusing, and surface pseudomembrane formation (PSM) was evident along the glomerular tuft endothelium of *Ampd2* ^m/m^ mice (white arrowheads). In addition to a minimal to moderate thickening of the basal lamina (GBM), there was a subtle increase in cellularity of the mesangial matrix (MM) and an increase in inflammatory cells, predominantly lymphocytes (LC) in *Ampd2* ^m/m^ mice. RBC = Red Blood Cell. Upper photomicrographs 4200×; lower photomicrographs 6000×; Bar = 2 μm.

To confirm the effect of the A341S mutation in the kidneys of *Ampd2*^m/m^ mice we investigated Ampd2 protein abundance in both strains and verified that Ampd2 protein was absent in the liver and kidney of pure B6 *Ampd2*^m/m^ mice (Figure 6A). NS was verified in *Ampd2*^m/m^ mice by kidney histology (Figure 6B). Proteinaceous tubular casts and interstitial nephritis were observed in *Ampd2*^m/m^ mice and not *Ampd2*^+/+^ mice (Figure 6B-D). By TEM, glomeruli of selected *Ampd2*^m/m^ mice and *Ampd2*^+/+^ mice were examined to ascertain any ultrastructural differences (Figure 7). Glomeruli from *Ampd2*^m/m^ mice showed effacement, fusion, and blebbing of podocyte foot processes, minimal to moderate thickening of the glomerular basement membrane, as well as denuding with subsequent pseudomembrane formation of the glomerular endothelium. While damage to foot processes, basal lamina and glomerular endothelium represents impairment of the glomerular filtration membrane, the subtle increase in cellularity of the mesangium, along with a predominant lymphocytic inflammatory infiltrate seen in *Ampd2*^m/m^ mice, further supports changes secondary to glomerular dysfunction with ensuing proteinuria.

### Effect of a potent AMPD/adenosine deaminase inhibitor on lipid and renal profiles of normal mice

Deoxycoformycin is a potent, but nonspecific inhibitor of AMPD/adenosine deaminase and is an approved chemotherapeutic drug. It dramatically decreases nucleotide metabolism and affects the cells ability to process DNA [15]. B6 mice were given a single injection of 10 mg/kg deoxycoformycin to determine the effect on plasma cholesterol and urinary protein levels. At 24 hours post injection, a rise in cholesterol as well as protein urea was seen in the plasma of deoxycoformycin treated mice (Additional file 2: Figure S1A and S1B). This data could suggest a link between dyslipidemia and NS when Ampd is inhibited, though the possibility that the response was an acute health reaction cannot be overlooked.

## Discussion

In the present study, we identified a mutation in the *Ampd2* gene that leads to NS and hypercholesterolemia. The combined phenotypes have not previously been reported in *Ampd2* knockout mice [14]; minimal change kidney disease was observed in these mice, and it was speculated that Ampd2 function was critical for podocyte survival and related to defects in Inosine-5′-monophosphate (IMP). Furthermore, despite dyslipidemia and minimal change disease being commonly associated in human NS, our *Ampd2*^m/m^ mice offer the first description of a mouse model to present both phenotypes. Further investigation will be needed to elucidate if these phenotypes are physiologically connected, or result independently from Ampd2 tissue-specific deficiency. Towards this end, restoring Ampd2 function in the liver or kidney of the *Ampd2*^m/m^ mice could allow dissection of this relationship.

We identified a transient cholesterol phenotype in mixed B6/C3 *Ampd2*^m/m^ mice, and have now confirmed that the renal phenotype is transient as well, temporally mirroring the cholesterol levels. In successive generations of the mixed B6/C3 *Ampd2*^m/m^ mice we noticed less severe incidence of hypercholesterolemia and speculate that this could be due to the genetic drift of modifier alleles in the mixed B6/C3 background. To address this hypothesis, we generated pure backgrounds of *Ampd2*^m/m^ mice on both B6 and C3 backgrounds. The pure B6 *Ampd2*^m/m^ mice do have a penetrant but lesser phenotype for the cholesterol levels and a stronger phenotype for the renal parameter (Figure 5). Conversely, pure C3 *Ampd2*^m/m^ mice showed no phenotype (data not shown). Together, this suggests that the mixed background can lead to a more severe phenotype, supporting the potential presence of modifier alleles and their epistatic effects on lipid traits [16].

The renal phenotype in the B6 *Ampd2*^m/m^ mice was further characterized by TEM. The kidneys were collected when lipid and renal phenotypes were most severe. Consistent with the proteinuria observed in the urine and proteinaceous tubular casts seen on histopathology, *Ampd2*^m/m^ mice were found to have damage to the glomerular filtration membrane at the ultrastructural level. Increases in inflammatory cells and in cellular debris also indicated progressive renal disease in *Ampd2*^m/m^ mice.

It is of interest to understand how the loss of Ampd2 function led to alterations in lipid profiles and kidney function. Ampd plays roles both in the purine nucleotide cycle as well as the catabolism of AMP to uric acid. In the purine nucleotide cycle, three enzyme catalyzed reactions convert AMP to fumarate, in order to increase the concentration of TCA cycle intermediates [17]. The generation of fumarate provides skeletal tissue with its only source of intermediate substrate for the TCA cycle [18] and loss of Ampd function reduces fumarate production via the purine nucleotide cycle. This effect could result in changes in the concentrations of malate, oxaloacetate, citrate, isocitrate, α-ketoglutarate and succinate in the TCA cycle. Furthermore in the *Ampd2*^m/m^ mice, malate and citrate levels were elevated while succinate levels were suppressed (Additional file 1: Table S1). These alterations in TCA cycle intermediates are not consistently decreased as could be expected by a defective purine nucleotide cycle by loss of Ampd2, suggesting that this aspect of hepatic Ampd2 does not underpin the lipid phenotype of the *Ampd2*^m/m^ mice.

Ampd is also the rate limiting step in the degradation of AMP [19], a defect in Ampd would be expected to lead to an increase in AMP and a decrease in any intermediates downstream of AMP, including IMP, inosine, hypoxanthine, xanthine and uric acid. The metabolomic data showed significant reduction in the downstream uric acid catabolic intermediate, allantoin (Additional file 1: Table S1). If the hepatic deficiency of Ampd2 is the primary cause of the dyslipidemia, the defect in purine catabolism as here could provide a pathogenic mechanism. Along these lines Yang et al. [20] have reported that allantoin administration in DIO mice significantly improved dyslipidemia. Metabolomic analysis also demonstrated significant increases in plasma plant sterols including campesterol and β-sitosterol. These dietary derived sterols are eliminated from the body through hepatic biliary secretion using the ATP binding cassette G5/8 (ABCG5/8) transporter complex. No gene expression changes were observed for ABCG8 (Figure 3A) suggesting the accumulation of β-sitosterol and campesterol were due to defects in posttranslational ABCG5/8 transporter function. Thus, one mechanism that may lead to elevated plasma cholesterol observed in *Ampd2*^m/m^ mice may be defects in ABCG5/8 transporter function.

The presence of dyslipidemia together with kidney abnormalities would normally be indicative of NS as the primary defect in the *Ampd2*^m/m^ mice, as urinary protein loss is known to stimulate an increase in LDL synthesis by the liver [21]. However, the *Ampd2*^m/m^ mice do not exhibit significantly elevated triglycerides (Figure 2C), a hallmark of dyslipidemia secondary to NS due to impaired clearance of chylomicrons and VLDL [21]. Furthermore induction of hypercholesterolemia during NS is primarily related to upregulation of HMG-CoA reductase activity [22]. An upregulation of *Hmgcr* was not observed in the liver of *Ampd2*^m/m^ mice, and in fact, a trend of decreased gene expression was observed (Figure 3). Insulin-induced gene 1 (*Insig1*) was decreased by 56% in *Ampd2*^m/m^ mice, possibly resulting in less binding of HMG CoA Reductase and increased cholesterol biosynthesis. Transcript levels of 3-hydroxy-3-methylglutaryl-CoA synthase (*Hmgcs*) were 47% lower in *Ampd2*^m/m^ mice, possibly due to feedback inhibition caused by high levels of circulating plasma cholesterol, albeit only a minimal reduction in *Srebp1* and *2* was observed. It has also been reported that rats with NS accompanied with hypercholesterolemia lack *Cyp7a1* upregulation, while rats that have equally high hypercholesterolemia due to a high fat diet do not present with NS and show marked elevation of *Cyp7a1* RNA levels [22, 23]. As shown in Figure 3, *Ampd2*^m/m^ mice demonstrated a modest but significantly increased expression of *Cyp7a1* mRNA, consistent with the dietary and not the NS induced lesion. This may be a compensatory mechanism to enhance cholesterol disposal through bile acid synthesis and secretion and switching to a different transporter in response to a defect in ABCG5/8 function. Other discrepancies between NS and dyslipidemia in *Ampd2*^m/m^ mice and other rodent NS models exist. Zhou et al. [24] as well as others [25, 26] have reported that Ldlr protein abundance is significantly reduced in NS rats, with no concurrent reduction in *Ldlr* gene expression, whereas *Ampd2*^m/m^ mice showed significant reductions in both. Shearer et al. [27] reported that in the nephrotic rat, proteinuria resulted in reduced HDL ApoE content. We found increases in both HDL-C and ApoE levels in our *Ampd2*^m/m^ mice. Liu and Vaziri [28] report that NS induced LDLR deficiency is due to upregulation of PCSK9 and IDOL, both post-translational regulators of LDLR. We see no increased expression of either gene in the *Ampd2*^m/m^ mice. Collectively, these data suggest that dyslipidemia is a direct consequence of Ampd2 deficiency, and not a consequence of NS. We propose that the pathogenic mechanism relates to defective catabolism of AMP to uric acid, including allantoin, versus impairment in the purine nucleotide cycle.

Recently, Akizu et al. [29] reported an early onset neurodegenerative condition resulting from mutations in *AMPD2* and found that AMPD2 plays a critical role in the maintenance of guanine nucleotide pools in relation to adenosine derivatives on de novo purine synthesis. No neurological phenotypes were observed in *Ampd2* knockout mice. Furthermore only *Ampd2/3* double knockout mice exhibited slightly reduced brain size, and showed little evidence of the neuronal loss observed in the *AMPD2* mutant human population. Nucleotide analysis of the double knockout brains showed increases in ATP nucleotide levels and a decrease in guanosine triphosphate, consistent with the increased ATP found in the liver of our *Ampd2*^m/m^ mice. We also confirm a significant reduction in levels of guanine in *Ampd2*^m/m^ mice; however our mice exhibited no obvious neurological phenotype.

In conclusion, we have identified a novel mouse model of transient hypercholesterolemia with concurrent NS associated with defective Ampd2 function. Ascertaining whether the two phenotypes are interrelated and the molecular mechanism of Ampd2 loss and hypercholesterolemia will require further investigation.

## Materials and methods

### Mouse breeding

Founder mice were identified at McLaughlin Research Institute [11] and subsequent generations were maintained at Charles River Laboratories to generate mixed C57BL/6 J (B6) C3.SW-*H2*^b^/SnJ (C3) animals. The *Ampd2* line was backcrossed to B6 via MAX-BAX® technology, and 2 of the 100% B6 congenic animals were obtained for further heterozygous × heterozygous mating. Mice were cared for in accordance to the *Guide for the Care and Use of Laboratory Animals, 8*^*th*^ Edition. Animals were single housed at an AAALAC accredited facility in non-sterile ventilated micro-isolator housing on corn cob bedding. All research protocols were approved by the (Amgen, Inc; Thousand Oaks) Institutional Animal Care and Use Committee. Animals had *ad libitum* access to pelleted feed (diet 2020X, Harlan Teklad; Indianapolis, IN) and water (reverse osmosis chlorinated water) via automatic watering system. Animals were maintained on a 12:12 hour light: dark cycle in rooms at 20–26°C, humidity maintained between 30-70% and had access to enrichment opportunities.

### Plasma and urine collection and analysis

Blood samples were collected in EDTA plasma tubes via the retro-orbital sinus of conscious mice. Plasma total cholesterol, HDL-cholesterol, triglyceride and albumin levels were measured using the Olympus AU400e Chemistry Analyzer (Olympus America, Inc; Center Valley, PA) from plasma thawed on ice and diluted four-fold with saline. ApoA1 and ApoE values were determined by immunoassay (Linco Research; St. Charles, MO). Proteinuria was measured by dipping Albustix reagent strips directly into urine (Siemens Medical; Malvern, PA).

### Deoxycoformycin studies

Commercial deoxycoformycin (Pentostatin; Tocris Bioscience; Bristol, UK) was dissolved in saline at a concentration of 25 mg/mL. Subsequent dilutions were made with saline and the mice were dosed via intraperitoneal injections at a concentration of 10 mg/kg. Baseline plasma samples were collected at 9 AM, immediately preceding a single dose of deoxycoformycin, and terminal plasma samples were collected 24 hours later.

### Quantitative RT-PCR analyses

Total liver RNA was isolated using an RNeasy Mini kit (QIAGEN GmBH; Hilden, Germany) and A_260_ was measured using a Beckman Coulter DU 800 spectrophotometer. RT-PCR reactions were set up using QuantiTect Multiplex RT-PCR Kit (QIAGEN), and 100 ng RNA per reaction was used, with appropriate oligonucleotides and probes; Cyclophilin A used as the housekeeping gene (sequences available upon request). Reactions were run on an ABI Prism 7900HT temperature cycler (Life Technologies; Carlsbad, CA). Results were analyzed using SDS2.2.2 software (Life Technologies).

### Lipoprotein analysis by fast performance liquid chromatography (FPLC)

Size-exclusion chromatography was performed by incorporating two Superose 6 10/300 GL columns (Amersham Biosciences; Piscataway, NJ) in tandem as described [30]. Fraction volumes of 275 μL were collected per well, from which 80 μL was removed and mixed with 120 μL of reagent from a colorimetric Cholesterol E Kit (Wako Chemicals USA; Inc., Richmond, VA) to measure cholesterol levels. To ensure linearity, a standard curve of pure cholesterol was included for each sample plate.

### Metabolomics

Mass spectrometry-based metabolite profiling analysis was performed at Metanomics Health GmBH (Berlin, Germany) on plasma and liver samples processed and extracted using a proprietary methods yielding a lipid and polar fraction which were used for gas chromatography–mass spectrometry (GC-MS) and liquid chromatography-tandem mass spectrometry (LC-MS/MS) analysis, respectively. For GC-MS analysis the samples were sequentially derivatized before measurement. For LC-MS/MS analysis a proprietary technology was applied which allows target and high sensitivity Multiple Reaction Monitoring profiling in parallel to full screen analyses. The method has been validated in separate experiments to determine intra- and interday variability, blank portions and linearity of each metabolite comprising known and unknown signals. Thresholds for reliable determination of the metabolite have been evaluated and confirmed within the study. Liver metabolite data were normalized to the dry weight of samples. For plasma samples, this step was not necessary due to the application of the same volume for each plasma sample. Each metabolite was normalized against the median of the data obtained from the control animals (ratios *vs.* control). Two hundred and twenty four metabolites were measured in plasma, 152 metabolites with known structure. For liver samples, 574 metabolites were measured, 222 metabolites with known structure. Metabolites with statistically significant differences between genotypes are presented in Additional file 1: Table S1.

### High performance liquid chromatography (HPLC) analysis of AMP, ADP, and ATP

Frozen (-80°C) mouse liver samples were homogenized at 10% (w/v) in acetonitrile, 10 mM KH_2_PO_4_ buffer pH 7.4 (3:1). The homogenate was centrifuged at 20,000 × g, 4°C, for 10 minutes and the supernatant centrifuged again at 20,000 × g, 4°C, for 10 minutes. The acetonitrile was removed by chloroform extraction three times. Final aqueous layer extract was diluted with 10 mM KH_2_PO_4_ Buffer, pH 7.4 to initial volume. Samples (20 μL) were analyzed using an Agilent 1100 Series HPLC equipped with a Hypersil Gold C-18, 250 × 4.6 mm 5 μm particle size HPLC column at a flow rate of 0.75 mL/minute using a 130 min step gradient protocol as follows: 10 minutes (100% A-10 mM tetrabutylammonium hydroxide, 10 mM KH_2_PO_4_, 0.125% methanol, pH 7.0), 3 minutes (80% A: 20% B- 2.8 mM tetrabutylammonium hydroxide, 100 mM KH_2_PO4, 30% methanol, pH 5.5), 10 minutes (70% A: 30% B), 12 minutes (55% Line A: 45% B), 11 minutes (40% A: 60% B), 9 minutes (35% A: 56% B), 10 minutes (25% A: 75% B), 30 minutes (100% B), 36 minutes (100% A). Spectra were collected at 206 nm and 260 nm for AMP (29.4 Retention Time), ADP (41.4 Retention Time), and ATP (49.1 Retention Time). Data was processed using Agilent Chemstation software using a standard curve for each analyte. The precision (%CV) of the assay was very good: 3.9% for AMP, 6.3% for ADP, and 3.9% for ATP with a limit of quantification of 0.1 μmol/g liver tissue.

### Gene sequencing

PCR DNA fragments of candidate genes were subcloned into the pCR II vector using a TA cloning kit and sequenced with M13F and M13R primers or directly sequenced with the PCR primers on an ABI 3730xl DNA Analyzer. To genotype the guanine to thymine transversion of *Ampd2*, a *Bse*R1 restriction digest was applied to a 173 bp PCR product amplified with the oligonucleotides 5′-GCACCTGCAGTCCTCATGTTTGTATAGC-3′, and 5′-GCTTGATGAAGCGCAGTAGATGTTTCTGG-3′.

### Protein analysis

Approximately 20–40 mg of frozen liver or kidney were homogenized in NP40 lysis buffer with 1X of Complete Protease Inhibitors Cocktail (Roche; Indianapolis, IN). Protein lysates were resolved on 4-12% Bis-Tris polyacrylamide gels (Life Technologies). Separated proteins were transferred to PVDF membranes (Life Technologies), and blocked with Pierce Superblock. The blots were hybridized with antibodies directed against Ampd2 (QQ13; Santa Cruz Biotechnology, Inc. Dallas, TX), Ldlr (Cayman Chemical; Ann Arbor, MI), Gapdh (IMGENEX; San Diego, CA) or β-actin (Sigma-Aldrich) overnight at 4°C with gentle shaking. The membranes were washed 3 times with TBS-T, incubated with secondary antibody conjugated to HRP at room temperature for 1.5 hours, and washed another 3 times in large volume changes of TBS-T. The blots were processed with SuperSignal West Dura substrate (Thermo Fisher Scientific, Inc. Rockford, IL), and visualized on a Fluorochem HD2 imager (ProteinSimple, Santa Clara, California).

### Histological analysis

Samples of kidney were fixed by immersion in 10% neutral buffered formalin for 24 hours, trimmed into cassettes, processed routinely to paraffin embedment, sectioned at 5 μm, stained with hematoxylin and eosin and periodic acid methenamine silver [31] and examined histologically.

### Electron microscopy

A portion of the renal cortex was collected for transmission electron microscopy (TEM), minced into 1 mm cubes and placed into 2.5% glutaraldehyde fixative. Following fixation, the tissues were processed for TEM. Samples were first rinsed in 0.1 M Sorenson’s Phosphate buffer, post-fixed in 2% osmium tetroxide, embloc stained with uranyl acetate, after which the tissues were dehydrated through a graded series of alcohol and acetone then 100% Spurr’s resin and cured at 65°C. Semi-thin sections (500 nm sections stained with Toluene blue) of each resin block were examined and four resin blocks/animal were chosen for thin sectioning (80–90 nm, gold sections placed on a 100 μm mesh copper grid). The four grids per animal were examined on both a JEM 100 kV (JEOL USA; Inc. Peabody, MA) and a Tecnai 110 kV (TEI; Hillsboro, OR) microscope with ten digital photomicrographs shot for each resin block thereby providing an adequate number of photomicrographs for comparison between the genotypes.

### Statistical analysis

In some graphs individual mouse values are shown with means ± SEM. In other graphs only the means ± SEM are displayed. Statistical analyses were carried out by one-way ANOVA using a Tukey post test comparing with three or more groups. For statistical analyses comparing two groups unpaired two-tailed *t*-tests for used; * p <0.05; ** p <0.01; *** p <0.001; **** p <0.0001 *vs. Ampd2*^+/+^.

## Electronic supplementary material

Additional file 1: Table S1: Metabolomic analysis of significantly altered metabolites in liver and plasma in *Ampd2* ^m/m^ and *Ampd2* ^+/+^ mice. Values represent the fold change. Statistical analyses were carried out by unpaired two-tailed *t*-tests. (DOCX 28 KB)

Additional file 2: Figure S1: Effects of deoxycoformycin, a potent inhibitor of AMPD/adenosine deaminase in B6 mice. Cholesterol (A) as well as urinary protein levels (B) were elevated 24 hours post injection. Statistical analyses were carried out by unpaired two-tailed *t*-tests; * p <0.05 ***; p <0.001 *vs.* saline. (PDF 73 KB)
