# Supplementary material for: A mutation in Ampd2 is associated with nephrotic syndrome and hypercholesterolemia in mice
Source: Lipids Health Dis. 2014 Oct 31;13:167. doi: 10.1186/1476-511X-13-167 (PMC4232700; doi:10.1186/1476-511X-13-167)
Supplement: Supplementary file 1 — Additional file 1: Table S1: Metabolomic analysis of significantly altered metabolites in liver and plasma in Ampd2 m/m and Ampd2 +/+ mice. Values represent the fold change. Statistical analyses were carried out by unpaired two-tailed t-tests. (DOCX 28 KB) [file 12944_2014_1148_MOESM1_ESM.docx]

Table S1. Metabolite levels in liver and plasma in *Ampd2*^m/m^ and *Ampd2*^+/+^ mice. Values represent the fold change *vs*. +/+. Statistical analyses were carried out by unpaired two-tailed *t*-tests.

| Metabolite | Liver | | Plasma | |
| --- | --- | --- | --- | --- |
|  | *Ampd2*^+/+^ | *Ampd2*^m/m^ | *Ampd2*^+/+^ | *Ampd2*^m/m^ |
| **Amino Acids** |  |  |  |  |
| Alanine | 0.98 | 0.75^a^ | 0.98 | 0.91 |
| Histidine | 1.00 | 0.64^b^ | 1.01 | 0.78^b^ |
| Isoleucine | 1.06 | 0.95 | 0.96 | 0.76^a^ |
| Leucine | 1.04 | 0.97 | 0.99 | 0.70^c^ |
| Phenylalanine | 1.04 | 0.97 | 1.02 | 0.84^a^ |
| Tryptophan | 1.04 | 0.93 | 1.05 | 0.64^b^ |
| Valine | 1.04 | 0.93 | 0.97 | 0.77^a^ |
| **Amino Acid derivatives** |  |  |  |  |
| Indole-3-lactic acid | 0.93 | 0.78 | 1.02 | 0.69^a^ |
| **Carbohydrates and related** |  |  |  |  |
| Mannose | 1.09 | 1.12 | 1.03 | 1.35^b^ |
| myo-Inositol | 0.97 | 1.52^b^ | 1.03 | 1.32^a^ |
| **Fatty Acids** |  |  |  |  |
| 14-Methylhexadecanoic acid | 1.03 | 1.15 | 0.98 | 1.17^a^ |
| 16-Methylheptadecanoic acid | 0.96 | 1.11 | 1.03 | 2.14^c^ |
| Arachidonic acid (C20:cis[5, 8, 11, 14]4) | 1.02 | 1.22^a^ | 0.99 | 1.83^c^ |
| Conjugated Linoleic acid (C18:trans[9,11]2) (minor: conjugated Linoleic acid (C18:cis[9]trans[11]2)) | 0.95 | 0.81 | 1.03 | 1.32^a^ |
| Docosahexaenoic acid (C22:cis[4,7,10,13,16,19]6) | 0.97 | 1.21^b^ | 0.99 | 2.03^c^ |
| Eicosanoic acid (C20:0) | 0.89 | 0.66^a^ | 0.93 | 1.16 |
| Eicosaenoic acid (C20:1) | 1.01 | 0.54^b^ | NA | NA |
| Eicosapentaenoic acid (C20:cis[5,8,11,14,17]5) | NA | NA | 1.02 | 1.39^a^ |
| gamma-Linolenic acid (C18:cis[6,9,12]3) | 0.98 | 1.16 | 1.02 | 2.56^c^ |
| Heptadecanoic acid (C17:0) | 1.03 | 1.13 | 1.03 | 1.76^c^ |
| Lauric acid (C12:0) | 0.96 | 1.11 | 1.06 | 1.64^a^ |
| Lignoceric acid (C24:0) | 0.99 | 1.10 | 1.08 | 1.45^a^ |
| Linoleic acid (C18:cis[9,12]2) | 1.02 | 1.00 | 1.02 | 1.95^c^ |
| Linolenic acid (C18:cis[9,12,15]3) | 0.96 | 0.70 | 1.03 | 1.57^a^ |
| Nervonic acid (C24:cis[15]1) | 1.00 | 1.34^c^ | 1.03 | 1.77^c^ |
| Oleic acid (C18:cis[9]1) | 1.00 | 0.56^a^ | 0.99 | 1.32 |
| Palmitic acid (C16:0) | 0.93 | 0.77 | 0.99 | 1.67^c^ |
| Palmitoleic acid (C16:cis[9]1) | 0.96 | 0.53^b^ | 1.00 | 1.18 |
| Stearic acid (C18:0) | 1.01 | 1.04 | 0.98 | 1.52^c^ |
| Tricosanoic acid (C23:0) | 0.99 | 1.26^a^ | 1.05 | 1.43^b^ |
| **Energy Metabolism and Related** |  |  |  |  |
| AMP | 1.05 | 1.45^a^ | NA | NA |
| Citrate (minor: Isocitrate) | 1.06 | 2.03^a^ | 1.03 | 1.04 |
| Fumarate | 0.97 | 1.17^a^ | 0.97 | 0.78^a^ |
| Lactate | 1.04 | 0.65^a^ | 0.98 | 1.04 |
| Malate | 1.02 | 1.58^c^ | 1.01 | 1.14 |
| Succinate | 0.92 | 0.66^a^ | 0.97 | 0.97 |
| **Nucleobases and Related** |  |  |  |  |
| 7-Methylguanine | 1.01 | 1.36^a^ | NA | NA |
| Allantoin (minor: 5-Aminohydantoin) | 0.99 | 0.68^a^ | 0.94 | 0.87 |
| Guanine | 0.98 | 0.86^a^ | NA | NA |
| **Cholesterol, Fatty Alcohols and Related** |  |  |  |  |
| Cholesterol | 1.03 | 1.20^a^ | 0.99 | 1.56^b^ |
| Cholesterol, total | 1.00 | 1.32^b^ | NA | NA |
| Cholesterylester C18:2 | 0.95 | 3.85^b^ | NA | NA |
| Cholesterylester C20:4 | 0.94 | 5.31^a^ | NA | NA |
| Dihydrocholesterol | 1.01 | 1.45^c^ | NA | NA |
| Dodecanol | 1.04 | 1.48^a^ | 0.96 | 1.61 |
| Glycerol, lipid fraction | 1.02 | 0.63^b^ | 1.06 | 1.57 |
| Hentriacontane | 0.99 | 1.27^c^ | NA | NA |
| Pentadecanol | 0.96 | 0.60^b^ | NA | NA |
| **Glycerides Mono-, Di-, Triglycerides** |  |  |  |  |
| DAG (C18:1, C18:2) | 0.99 | 1.03 | 0.96 | 1.64^c^ |
| TAG (C16:0, C16:1) | 1.00 | 0.28^c^ | 0.95 | 0.82 |
| TAG (C16:0, C18:2) | 0.93 | 0.46^b^ | 1.00 | 1.36 |
| TAG (C18:1, C18:2) | 0.93 | 0.55^a^ | 1.00 | 1.61^b^ |
| TAG (C18:2, C18:2) | 0.99 | 0.84 | 0.99 | 1.96^b^ |
| TAG (C18:2, C18:3) | NA | NA | 1.01 | 2.07^a^ |
| **Glycolipids** |  |  |  |  |
| myo-Inositol, lipid fraction | 0.99 | 1.01 | 1.01 | 1.32^c^ |
| **Phospholipids** |  |  |  |  |
|  |  |  |  |  |
| Glycerol phosphate, lipid fraction | 0.92 | 1.15 | 1.00 | 1.44^c^ |
| Lysophosphatidylcholine (C16:0) | 0.97 | 1.10^a^ | 1.02 | 1.01 |
| Lysophosphatidylcholine (C17:0) | 1.00 | 1.34^b^ | 1.00 | 1.46^c^ |
| Lysophosphatidylcholine (C18:0) | 1.01 | 1.21^c^ | 1.00 | 1.04 |
| Lysophosphatidylcholine (C20:4) | 1.03 | 1.11 | 1.00 | 0.96^a^ |
| myo-Inositol-1-phosphate, lipid fraction (myo-Inositolphospholipids) | 1.02 | 1.13^a^ | 0.99 | 1.44^b^ |
| myo-Inositol-2-phosphate, lipid fraction (myo-Inositolphospholipids) | 1.00 | 1.15^a^ | 0.98 | 1.34^c^ |
| Phosphate, lipid fraction | 1.05 | 1.36^b^ | 0.98 | 1.47^c^ |
| Phosphatidylcholine (C16:0, C16:0) | 1.00 | 1.19^b^ | 1.00 | 0.98 |
| Phosphatidylcholine (C16:1, C18:2) | 0.99 | 1.11^a^ | 0.97 | 1.05^a^ |
| Phosphatidylcholine (C18:1, C18:2) (minor:Phosphatidylcholine (C16:0, C20:3) | NA | NA | 1.00 | 1.03^b^ |
| **Sphingolipids** |  |  |  |  |
| 3-O-Methylsphingosine (minor: Sphingolipids, erythro-Sphingosine, threo-Sphingosine) | 1.01 | 1.27^a^ | 1.00 | 1.58^c^ |
| 5-O-Methylsphingosine (minor: Sphingolipids, erythro-Sphingosine, threo-Sphingosine) | 1.01 | 1.28^b^ | 1.01 | 1.64^c^ |
| Ceramide (d18:1, C24:0) | 1.00 | 0.92 | 1.03 | 1.77^c^ |
| Ceramide (d18:1, C24:1) (minor: Ceramide (d18:2, C24:0) | 1.00 | 0.99 | 1.00 | 1.77^c^ |
| erythro-Dihydrosphingosine | 0.96 | 1.08 | 1.02 | 1.28^b^ |
| erythro-Sphingosine (minor: Sphingolipids) | 1.00 | 1.23^b^ | 0.99 | 1.46^c^ |
| Phytosphingosine | 0.99 | 0.95 | 1.00 | 1.22^a^ |
| Sphingomyelin (d18:1, C16:0) | 1.00 | 1.16^b^ | 1.00 | 1.00 |
| Sphingomyelin (d18:1, C24:0) | 0.97 | 1.08^a^ | 1.01 | 1.09 |
| Sphingosine-1-phosphate | 1.05 | 1.37^a^ | NA | NA |
| threo-Sphingosine (minor: Sphingolipids) | 1.00 | 1.20^a^ | 0.92 | 1.38^b^ |
| **Other hormones, signal substances and related** |  |  |  |  |
| Thyroxine (T4) | NA | NA | 1.08 | 0.63^b^ |
| **Vitamins, cofactors and related** |  |  |  |  |
| Ascorbic acid (minor: Glucose) | 1.01 | 0.74^a^ | 0.98 | 0.92 |
| Coenzyme Q10 | 1.04 | 1.13 | 1.13 | 2.07^b^ |
| Coenzyme Q9 | 1.01 | 1.06 | 1.00 | 1.63^c^ |
| Nicotinamide | 0.99 | 1.11^a^ | NA | NA |
| Opthalmic acid | 0.89 | 1.70^a^ | NA | NA |
| S-Adenosylhomocysteine | 0.99 | 0.73^a^ | NA | NA |
| Threonic acid | 1.05 | 1.90^b^ | 0.98 | 0.86 |
| **Miscellaneous** |  |  |  |  |
| Acetylpyruvate (minor: 2,5-Dioxovaleric acid, Itaconic acid) | 1.01 | 1.13^a^ | NA | NA |
| β-Sitosterol | 1.04 | 1.09 | 0.98 | 1.58^c^ |
| Campesterol | 1.00 | 1.12 | 1.01 | 1.91^c^ |
| Carnitine | 1.00 | 0.77^b^ | NA | NA |
| Creatinine (minor: Creatine, Phosphocreatine) | 0.93 | 1.40 | 1.02 | 1.63^a^ |
| Ethanolamine | 0.99 | 1.10^a^ | 1.00 | 1.03 |
| O-Phosphoethanolamine | 0.98 | 1.32^a^ | 0.99 | 1.08 |
| Phophate (inorganic and organic phosphate) | 0.97 | 1.11^a^ | 1.02 | 0.90 |
| Phosphocreatinine | 1.01 | 1.18^a^ | NA | NA |
| Putrescine (minor: Agmatine) | 0.97 | 1.44^a^ | NA | NA |
| Pyrophosphate (PPi) | 1.11 | 2.19^a^ | NA | NA |
| Sarcosine | 0.98 | 0.60^a^ | NA | NA |
| Spermidine | 1.00 | 1.42^c^ | NA | NA |

^a^ p<0.5 ^b^ p<0.01 ^c^ p<0.001 *vs Ampd2*^+/+^; NA = Not Applicable
