# Supplementary figures and images for: A mutation in Ampd2 is associated with nephrotic syndrome and hypercholesterolemia in mice
Source: Lipids Health Dis. 2014 Oct 31;13:167. doi: 10.1186/1476-511X-13-167 (PMC4232700; doi:10.1186/1476-511X-13-167)

Supplemental Figure 1.

A.

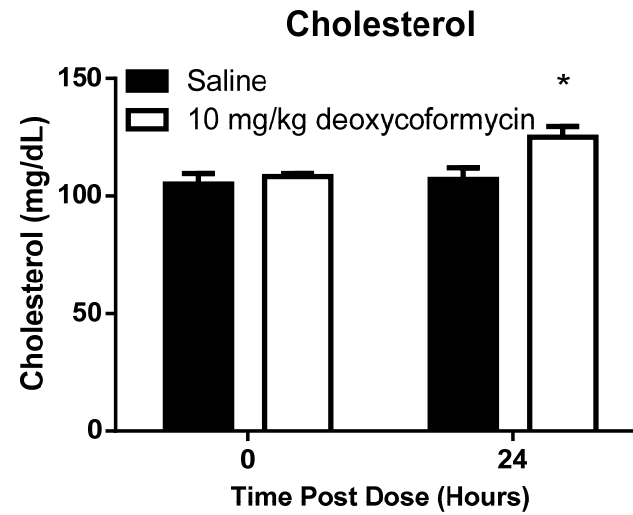

B.

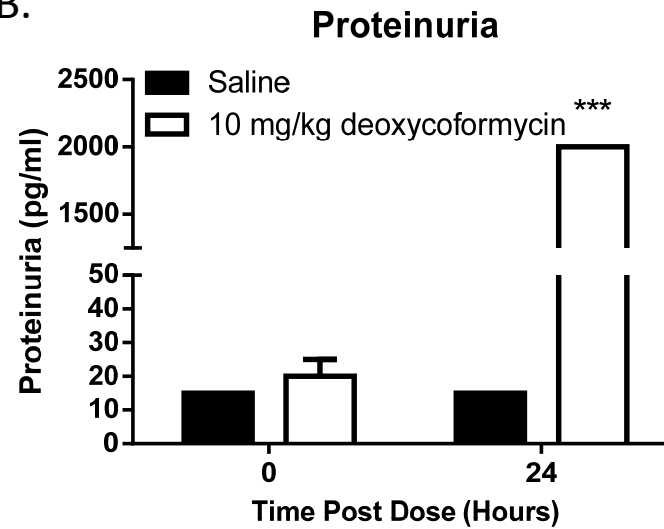

Supplement: Supplementary file 2 — Additional file 2: Figure S1: Effects of deoxycoformycin, a potent inhibitor of AMPD/adenosine deaminase in B6 mice. Cholesterol (A) as well as urinary protein levels (B) were elevated 24 hours post injection. Statistical analyses were carried out by unpaired two-tailed t-tests; * p <0.05 ***; p <0.001 vs. saline. (PDF 73 KB) [file 12944_2014_1148_MOESM2_ESM.pdf]
